# Interim Performance Progression (IPP) During Consecutive Season Best Performances of Talented Swimmers

**DOI:** 10.3389/fspor.2020.579008

**Published:** 2020-11-03

**Authors:** Aylin K. Post, Ruud H. Koning, Inge K. Stoter, Chris Visscher, Marije T. Elferink-Gemser

**Affiliations:** ^1^Center for Human Movement Sciences, University Medical Center Groningen, University of Groningen, Groningen, Netherlands; ^2^Department of Economics, Econometrics & Finance, Faculty of Economics and Business, University of Groningen, Groningen, Netherlands

**Keywords:** swimming, acquisition of expertise, talent development, performance progression analysis, elite athletes

## Abstract

**Objective:** The main goal of the present study was to investigate the interim performance progression (IPP) of talented swimmers. Part of this group ultimately made it to the top (referred to as elite swimmers) whereas others did not make it to the top (referred to as high-competitive swimmers). Rather than investigating performance progression based solely on season best performances, we included the first swim performance of the season in the metrics of IPP. Knowledge about the IPP of talented swimmers from and toward their season best performances relative to the first swim performance of the season will enhance our understanding of changes in season best performances during the talent trajectory and provide valuable insights for talent development and selection processes in competitive swimming.

**Methods:** Fifteen thousand nine hundred and forty four swim performances (first swim performances of the season and season best performances) between 1993 and 2019 of 3,199 talented swimmers (of whom 556 reached elite level and 2,643 reached high-competitive level) were collected from Swimrankings and related to the prevailing world record of the corresponding sex. The pattern of IPP was represented by two phases: phase A and phase B. Phase A reflected the performance progression between the previous season best performance and the first swim performance of the current season (PP_A_) and phase B reflected the performance progression between the first swim performance of the current season and the season best performance of the current season (PP_B_). Depending on the normality check, we used independent sample *t*-tests or Mann Whitney tests to establish significant differences in PP_A_ and PP_B_ between elite and high competitive swimmers per age category per sex (*p* < 0.05).

**Results:** Without denying individual differences, male elite swimmers improved more during phase B from age 15 till 24 compared to high-competitive swimmers (20.5% vs. 13.1%, respectively, *p* < 0.05). Female elite swimmers improved more during phase B from age 13 till 23 compared to high-competitive swimmers (21.1% vs. 14.6%, respectively, *p* < 0.05). Except for age 14 in males, there were no significant differences between performance groups in PP_A_.

**Conclusion:** Talented swimmers who ultimately made it to the top (elite swimmers) are characterized with different patterns of IPP compared to talented swimmers who did not make it to the top (high-competitive swimmers). After puberty, elite and high-competitive swimmers performed in general ~1% slower at the start of their season compared to their previous season best performance (PP_A_). However, elite swimmers improved more in the period between their first swim performance of the season and their season best performance (PP_B_) from age 13 (females) and age 15 (males) onwards.

## Introduction

For coaches and stakeholders in competitive swimming, season best performances and national rankings are the main information source for talent identification and selection processes (KNZB, 2018). Based on this information and their perception about how that information relates to future performance, they have to make decisions about whether or not a swimmer is selected for a talent development program (Schorer et al., 2017). However, several researchers are questioning this one-sided approach in which performance at early stages of development (e.g., age 12 onwards in competitive swimming; KNZB, 2018) is used as an indicator of future performance (Abbott et al., 2005; Vaeyens et al., 2009; Elferink-Gemser et al., 2011). They advocate that there are multiple pathways to reach expertise and that there is a risk to erroneously overlook athletes as being talented by focusing on current performance only (Vaeyens et al., 2008; Gulbin et al., 2013; Till et al., 2016).

In order to provide scientific-based knowledge about the value of early age performance in competitive swimming, Post et al. (2020) tracked down the junior-to-senior performance development of top-elite swimmers at the 100 m freestyle event. This research was based on the analysis of season best performances and provided support for both perspectives. The findings showed that (1) compared to each other, top-elite swimmers follow unique individual developmental pathways toward expertise and (2) compared to other performance groups, top-elite swimmers in general progressively outperform their elite, sub-elite and high-competitive swimmers of similar age from 12 years onwards.

In addition to examining group averages as in the research of Post et al. (2020), upper and lower limits of swimmers who have made it to the top can provide relevant insights as well. Stoter et al. (2019) used the upper limits of elite speed skating performance (slowest performance per age and per sex for those who later reached the elite level in this sport) to define performance benchmarks for future speed skaters. The results showed that the majority of talented male and female speed skaters who performed within the performance benchmarks at a younger age, did not make it to the top. These findings combined with previous results of Post et al. (2020) inspire to continue the investigation of youth performance. What characterizes the performance development of those who are considered as talented swimmers (e.g., perform within performance benchmarks) and do reach the top compared to their talented counter peers who do not reach the top?

Probably, the answer to this question may not be hidden in solely tracking season best performances. Although monitoring and modeling season best performances highly contributed to a deeper understanding of performance development to the swimming top (Stewart and Hopkins, 2000; Costa et al., 2011; Allen et al., 2014; König et al., 2014; Post et al., 2020; Yustrus et al., 2020), it would be interesting to include additional swim performances in mapping performance progression of talented swimmers. As such, scientific-based data about (1) the progression between a swimmer's previous season best performance and his first swim performances of the season and (2) the progression between a swimmer's first swim performance of the season and his current season best performance could provide meaningful information about the interim performance progression (IPP) during two consecutive season best performances.

Knowledge about IPP during consecutive season best performances of talented swimmers would enhance our understanding of changes in season best performances during the talent trajectory. In particular, this is the case when IPP is investigated from a retrospective perspective in which talented swimmers who made it to the top (elite swimmers) are compared to their talented counter peers who in the end did not make it to the top (high-competitive swimmers). In here, a longitudinal approach is necessary as the road to the top is long and often combined with large inter-individual differences between swimmers due to processes of growth and maturation (Kannekens et al., 2011; Malina et al., 2015; Elferink-Gemser et al., 2018). This would provide valuable and additional insights about the general and individual performance patterns of swimmers on their way to the top, which can be used to optimize talent development programs. As such, federations, coaches and swimmers would benefit from a more detailed guideline toward elite swimming performances and be able to set and monitor realistic and data-driven goals about the development of swim performances during a swimming season. Moreover, IPP may be an additional variable to select and monitor swimmers who have the potential to make it to the top.

However, to the best of our knowledge, a longitudinal, retrospective analysis of IPP of talented swimmers with the potential to make it to the elite level has not been conducted yet. Therefore, the main goal of this study was to longitudinally and retrospectively investigate the IPP during consecutive season best performances of talented swimmers. Part of this group ultimately made it to the top (referred to as elite swimmers) whereas others did not make it to the top (referred to as high-competitive swimmers). Given the fact that at some point during their career, elite swimmers outperformed their peers, we hypothesize that elite swimmers have higher IPP compared to swimmers who did not reach elite level (high-competitive swimmers).

## Materials and Methods

### Ethical Approval

All procedures used in the study were approved by the Local Ethical Committee of the University Medical Center Groningen, University of Groningen, The Netherlands (201900334) in the spirit of the Helsinki Declaration with a waiver of the requirement for informed consent of the participants given the fact that the study involved the analysis of publicly available data.

### Data Collection

The swimmers we selected for this study were international male and female swimmers with performance data on the 100 m freestyle long course event. We chose this event because it is considered as the key distance in competitive swimming. It has been on the Olympic program since 1904 (men) and 1912 (women) and is characterized with the largest number of world-wide participants. Moreover, competition starts from an early age on and the word-wide competition level is high for both male and female swimmers (FINA, 2019; Swimrankings, 2019).

Performance data (in terms of swim times) was obtained from Swimrankings (2019), a recognized public data source which records international swimming race results. Performance data were collected from 88 countries across different parts of the world including Africa, America, Asia, Australia and Europe. We collected all available 100 m freestyle long course results from Swimrankings' database, which initially resulted in 2,864,4481 observations between 1993 and 2019.

### Data Processing

For the purpose of the present study, we transformed the structure of the dataset. Starting with individual competition observations (each observation e.g., swim performance stored into a unique row), we restructured the dataset in individual season observations (two observations e.g., swim performances stored in one row). The two observations we stored in one row were the first swim performance of the swimming season and the best swim performance of the swimming season. All other performance data within the season were discarded from further analysis.

Performance data from the 1st of January 2008 till the 1st of January 2010 were excluded from analysis (we exclude full-body polyurethane swimsuits; Toussaint et al., 2002; Tiozzo et al., 2009; Tomikawa and Nomura, 2009). Swim performances over 180 s were excluded from analysis to ensure a representative dataset. Based on swim dates, performance data were classified in swimming seasons. Each swimming season officially starts on the 1st of September of a calendar year and ends on the 31st of August of the next calendar year (FINA, 2019). Swimmers were classified in age categories based on their age on the 31st of December of the swimming season (KNZB, 2018). Therefore, all ages mentioned in the present study refer to the age category in which a swimmer participated during the swimming season and not the calendar age of the swimmer.

### Defining Swim Performance and Performance Groups

The present study includes swim performances of multiple generations, necessitating the correction of evolution in a given sport (Stoter et al., 2019; Post et al., 2020). The method we used to correct for the evolution in competitive swimming was introduced by Stoter et al. (2019) in the sport of speed skating and later successfully used by Post et al. (2020) in the sport of competitive swimming. Swim performances were related to the prevailing world record (WR) or the fastest time in textile swimsuit of the corresponding sex. The prevailing WR is the official WR at the date the swimmer performed the swim time. WRs from 2008 or 2009 were replaced by the prevailing fastest time in textile swimsuit. The corrected swim time will be referred to as relative Swim Time (rST) and is presented as a percentage of the prevailing world record or fastest time in textile swimsuit. In this study, rST defines swim performance (see Equation 1).

(1) $$\begin{array}{r} \text{relative}\;\text{swim}\;\text{time}\;\text{(rST)}=\left(\frac{\text{swim}\;\text{time}}{\text{world}\;\text{record}}\right)*\text{100\%} \end{array}$$

Two performance levels were defined: elite and high-competitive. Each performance level was characterized by sex-specific limits to account for differences in competition level between males and females (elite males: best rST ≤ 103.9%; elite females: best rST ≤ 105.8%; high-competitive males: 103.9% < best rST ≤ 114.0%; high-competitve females: 105.8% < best rST ≤ 115.1%). The limits were calculated as the mean of 5 season best rST's for the 50th swimmer from either the 100 m freestyle performance FINA World Ranking Lists of 2015-2019 (FINA, 2019) or the 100 m freestyle performance National Ranking Lists of the Netherlands 2015–2019 (Swimrankings, 2019). The limits of the elite performance level were equal to the average of the season best rST's of the 50th male and female swimmer of the FINA World Ranking List 2015-2019. The limits of the high-competitive level were defined so that they represented the 50th male and female swimmer of the National Ranking List of the Netherlands.

We determined each swimmer's best performance group by allocating the best rST ever to one of the two performance levels, meaning that a swimmer either once or multiple times has reached this performance level at any age. For example, if a male swimmer has a best rST of 109.0%, his best performance level corresponds with the limits of the high-competitive performance group. Swimmers with a best rST ever outside the limits of the high competitive level (best rST > 114.0% for males and best rST > 115.1% for females) were excluded from further analysis.

### Inclusion Criteria

We included talented swimmers of which some swimmers ultimately made it to the top (elite swimmers) and others did not (high-competitive swimmers). The inclusion criteria were: (1) swimmers who had at least one swim performance in the age category of 22 years or older (males) or 20 years or older (females). Based on research of Allen et al. (2014) we suggest that this is in general the expected minimum age for swimmers to achieve their career best performances. To ensure a dataset representing the developmental pathway toward peak performance, we solely included (2) swim performances up to and including the swimmer's career best swim performance. Furthermore, we selected only those swimmers who (3) where between the 12 and 24 years old; (4) had performance data of at least two consecutive swimming seasons (5) had two observations within a swimming season and (6) had season best rST's within the performance benchmarks.

The performance benchmarks were taken as indicator for future performances toward elite level swimming. Therefore, swimmers performing within these performance benchmarks were in the present study considered as talented swimmers. The performance benchmarks were based on previous research of Post et al. (2020) and reflect the maximal season best rST for elite swimmers per age and per sex (see Supplementary Table 1). Performance benchmarks were set to be monotone, meaning that with every successive maximal season best rST lower than the previous, the benchmark will decrease toward the value of this season best rST, but with every successive maximal season best rST higher than the previous, the benchmark will remain at the same value.

Table 1 represents the male/female distribution and the number of observations (i.e., total rSTs) for each performance group included for analysis, with an average of 3.6 ± 2.0 observations per swimmer.

**Table 1:** Total number of swimmers (*N* = 3,199) and observations (*N* = 8,005) for each performance group specified by sex for the analysis on interim performance progression (IPP).

	**Males**		**Females**	
**Performance level**	**Individuals**	**Observations**	**Individuals**	**Observations**
Elite	196	638	360	1,062
High-competitive	1,279	3,085	1,364	3,220

*elite males: best rST ≤ 103.9%; elite females: best rST ≤ 105.8%; high-competitive males: 103.9% < best rST ≤ 114.0%; high-competitve females: 105.8% < best rST ≤ 115.1%.

### Defining Interim Performance Progression (IPP)

The concept of interim performance progression (IPP) is explained as the pattern of performance progression during two consecutive seasons relative to a common reference point. Therefore, the pattern of IPP is described by two phases: phase A and phase B.

**Figure 1:**
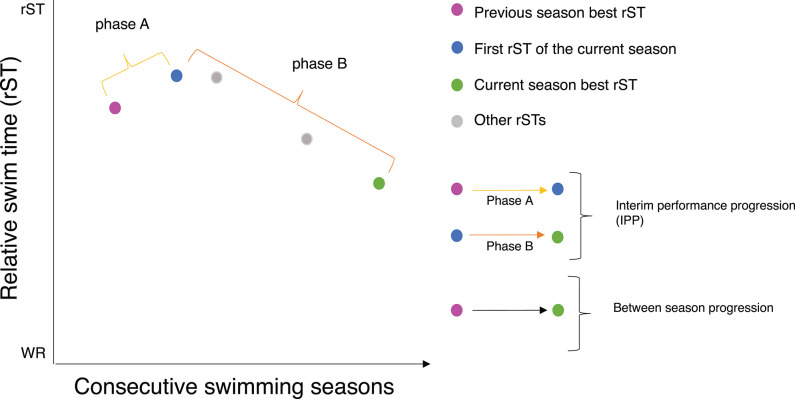
The concept of interim performance progression (IPP).

Phase A is presented as the period between the previous season best rsT and the first swim performance of the current season (first rST). Phase B is presented as the period between the first rST and the current season best rST. So, the first rST is the common reference point in phase A and phase B (see Figure 1). The first rST can be worse, the same or better than the previous season rST. In Figure 1, it is shown as worse. The current season best rST can be the same or better than the first rST. In Figure 1, it is shown as better. Ultimately, the current season best rST can be the worse, the same or better than the previous season best rST. In Figure 1, it is shown as better.

The performance progression during phase A (PP_A_) is defined as the percentage of the first rST relative to the previous season best rST (see Equation 2). This measure is constructed to reflect the start level of a swimmer relative to his best swim performance of the previous season. An outcome below the 100% means that the swimmer was faster than his previous season best rST (improved) and an outcome above the 100% means that the swimmer was slower than his previous season best rST (deteriorated). An outcome of 100% means that the swimmer is at the exact same level as his previous season best rST (stabilized).

(2) $$\begin{array}{r} PP_{A}=\left(\frac{\text{first}\;\text{rST}}{\text{previous}\;\text{season}\;\text{best}\;\text{rST}}\right)*\text{100\%} \end{array}$$

The performance progression during phase B (PP_B_) is defined as the percentage change a swimmer has moved toward the prevailing world record (see Equation 3). In other words, PP_B_ is relative to the gap a swimmer needs to close in order to break the prevailing world record or fastest time in textile swimsuit. PP_B_ reflects the difference between the best rST of the current season (current season best rST) and the first rST divided by the difference between the first rST and the prevailing world record or fastest time in textile swimsuit (see Equation 3).

A positive outcome indicated that a swimmer has moved toward the prevailing world record or fastest time in textile swimsuit and improved relative to his first rST. An outcome of 0% indicated that the swimmer's gap to the prevailing world record or fastest time in textile swimsuit stayed the same and that the swimmer did not improve relative to his first rST.

(3) $$\begin{array}{r} PP_{B}=-\left(\frac{\text{current}\;\text{season}\;\text{best}\;\text{rST}\;-\;\text{first}\;\text{rST}}{\text{first}\;\text{rST}-\text{100}}\right)*\text{100\%} \end{array}$$

As an example, we illustrate the pattern of IPP of a fictive swimmer with a season best rST of 106.5 in the previous season (2016/2017), a first rST of 107.6% in the current season (2017/2018) and a season best rST of 106.0% in the current season (2017/2018). His PP_A_ will be [107.6 (first rST)/106.5 (previous season best rST)^*^100%. In short his PP_A_ is (107.6/106.5)^*^ 100% = 101.0%. An outcome above the 100% means that the swimmer's SL is slower than his best rST of the previous season. His PP_B_ will be—[106.0 (current season best rST) − 107.6 (first rST)]/107.6 (first rST) − 100%. In short his PP_B_ is—(−1.6)/7.6 ^*^100 = 21%. A positive outcome indicates the swimmer moved toward the prevailing world record or fastest time in textile swimsuit and that he improved his swim performance between the start of the current season and the moment he swum his best rST of the current season. The pattern of IPP of this fictive swimmer is characterized by a small decrease in phase A (1% above his previous attained performance level), followed by an increase during phase B.

### Statistical Analysis

All data were analyzed for male and female swimmers separately using IBM SPSS Statistics 24 and R (R Core Team, 2019) (R version 3.6.0). Mean scores and standard deviations were calculated for swim performance (previous season best rST, first rST and current season best rST), performance progression in phase A (PP_A_) and performance progression in phase B (PP_B_) for the two performance groups per age category (see Supplementary Tables 2, 3). The normality of the distributions was assumed for *n* > 30, according to the central limit theorem (Field, 1993). For *n* < 30, distributions were visually inspected by histograms and Q-Q plots. Per age category, an independent-samples *t*-test (normality assumed) or Mann-Whitney test (normality violated) was conducted to compare PP_A_ en PP_B_ between elite and high-competitive swimmers. To interpret the scores, effect sizes (r of *d*, depending on normality) were calculated. An effect size of ∽0.20 (*d*) or 0.10 (r) was considered small, 0.50 (*d*) or 0.30 (r) moderate and 0.80 (*d*) or 0.50 (r) large (Cohen, 1969). Statistical tests were executed for the age categories in which there were more than six observations in the elite performance group. For all tests, *p* < 0.05 was set as significance.

## Results

**Figure 2:**
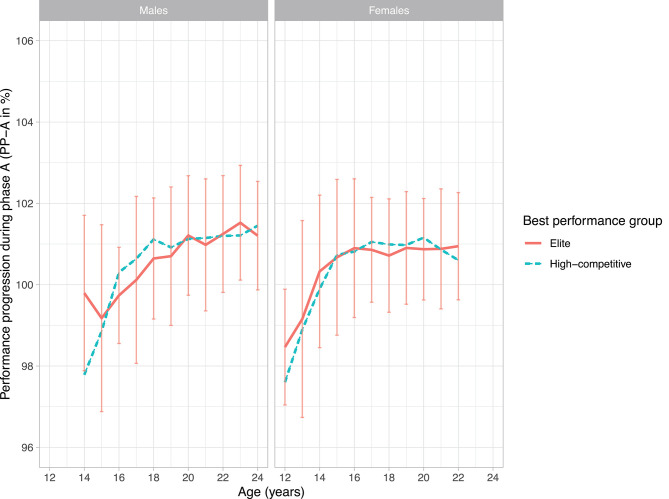
Performance progression in phase A (mean PP_A_) of male and female elite and high-competitive swimmers. Scores above the 100% indicate that the first rST is slower compared to the previous season best rST. For the purpose of this study, SDs are only shown for elite swimmers.

**Figure 3:**
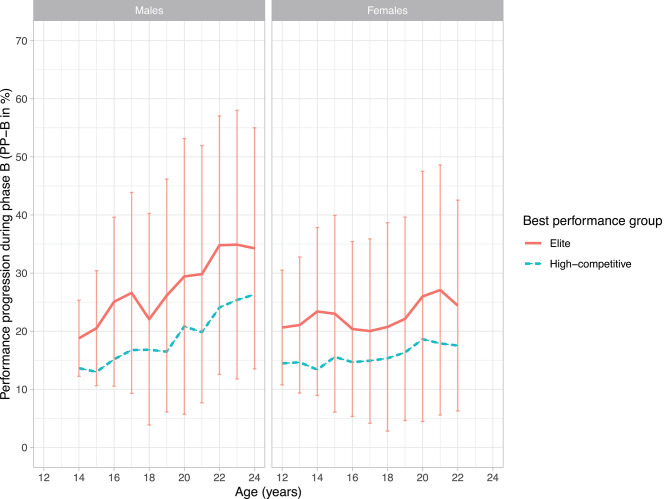
Performance progression in phase B (mean PP_B_) of male (right) and female (left) elite and high-competitive swimmers on the 100 m freestyle from age 12 to 24. Higher scores indicate higher progression. For the purpose of this study, SDs are only shown for elite swimmers.

Figures 2, 3 illustrate the performance progression in phase A (PP_A_) and phase B (PP_B_), respectively, of talented male and female swimmers on the 100 m freestyle from age 14 to 24 (males) and 12 to 22 (females). Within each age category, all swimmers performed within the corresponding performance benchmarks, however part of them reached the top (elite swimmers) and part of them did not reach the top (high-competitive swimmers). The average period of PP_A_ was 252 ± 87 days and the average period of PP_B_ was 102 ± 76 days.

Except for age 14 in males, Mann-Whitney tests and independent sample *t*-tests showed no significant differences between elite and high-competitive swimmers in PP_A._

For males, we found significant differences in PP_B_ between elite and high-competitive swimmers from age 15 till 24 (*p* < 0.05). From age 15 onwards, male elite swimmers improved on average more in their swim performance than male high-competitive swimmers in the period between their first swim performance of the current season and their current season best performance.

For females, we found significant differences in PP_B_ between elite and high-competitive swimmers from age 13 till 22 (*p* < 0.05). From age 13 onwards, female elite swimmers improved on average more in their swim performance than female high-competitive swimmers in the period between their first swim performance of the current season and their current season best performance. Corresponding test statistics are reported in Supplementary Tables 4, 5 (males and females, respectively).

## Discussion

The present study investigated the in interim performance progression (IPP) during consecutive season best performances of talented swimmers. Part of this group ultimately made it to the top (referred to as elite swimmers) whereas others did not make it to the top (referred to as high-competitive swimmers). The main findings of this study showed that without denying individual differences (1) elite swimmers improved more in swim performance than high-competitive swimmers during phase B (the period between the first rST and the current season best rST) and that (2) there were no differences between elite and high-competitive swimmers in performance progression between the previous season best performance and the first swim performance of the current season (PP_A_) (except for age 14 in males).

Considering these outcomes, it is important to notice that the results of the present study are inextricably linked to how we defined the metrics of IPP: PP_A_ and PP_B_. As it is well-known that at some point during a swimmers' career, the rate of performance progression begins to reduce (known as the principle of diminishing returns to training; Hoffman, 2014), we found it highly important to include metrics of IPP that enabled the interpretation of performance progression of swimmers relative to their previous performance level (PP_A_) and relative to the elite performance level (PP_B_). By relating performance progression to the gap a swimmer needs to close in order to break the prevailing world record or fastest time in textile swimsuit, PP_B_ accounted for the principle of diminishing returns and related performance progression to the (prevailing) fastest male or female swimmer of the world. Together, this makes that PP_B_ can be compared between swimmers of different performance levels and generations and simultaneously can function as measure to point out how much a swimmer moved forward to the prevailing world record or fastest time in textile swimsuit. In here, the present study aimed to make a more “fair” comparison between and within swimmers in a multigenerational and longitudinal dataset. To the best of our knowledge, the perspective on IPP and the related metrics of IPP have not been described in swimming literature yet.

Since IPP is explained as the pattern of performance progression during two consecutive seasons relative to a common reference point (first rST), the present study contributed to additional insights about the course of performance progression of talented swimmers. Descriptive statistics show that during puberty, talented male and female swimmers progress in the period between the previous season rST and the first rST (PP_A_) and in the period between the first rST and the current season best rST (PP_B_). In other words: they progressed in both phase A and phase B. However, post-puberty, progression during two consecutive season best performances generally took place in phase B rather than phase A. The latter suggests that coaches and swimmers should not get too discouraged if the first swim performance of the current season is ~1% slower compared to the previous season best performance.

As elite swimmers and high-competitive swimmers did not significantly differ in the performance progression in phase A (except for age 14 in males), we suggest that differences in PP_B_ between elite and high-competitive swimmers should not be accounted to previously emerged differences in PP_A_, but to different developmental patterns in phase B. Obviously, an intriguing question is: what causes these differences in developmental patterns and the higher PP_B_ of elite swimmers? In here, it is interesting to consider the inter-individual differences in adolescent growth processes and the quantity and quality of training hours as explaining factors (Ericsson et al., 1993; Malina et al., 2015). Moreover, differences in underlying performance characteristics between elite and high-competitive swimmers might relate to a larger performance potential (Elferink-Gemser et al., 2011). If so, PP_B_ might be a promising variable for talent development and selection processes as it may reflect this larger performance potential. However, the present study did not include any of these factors and consequently, more research is warranted. Therefore, a recommendation for future research would be to further unravel successful performance development to the top by tracking maturation, learning and training related to the personal performance characteristics of the individual swimmers (e.g., between 12 and 18 years) and their environment over time (Jonker et al., 2010; Elferink-Gemser and Visscher, 2013; Till et al., 2013). Moreover, as the present study showed large SDs within age categories and different effect sizes between age categories, it would be interesting to include multilevel modeling to examine within-subject variations and age-related effects in future studies investigating talented swimmers.

## Conclusion

The present study showed significant differences in IPP between talented swimmers who have made it to the top (referred to as elite swimmers) and talented swimmers who did not make it to the top (referred to as high-competitive swimmers). Without denying individual differences, talented swimmers who have made it to the top, improved more in the period between the first swim performance of the season and their current season best performance (PP_B_) than talented swimmers who did not make it to the top.

### Practical Implications

The findings of the present study can be used to compare interim performance progression (IPP) of talented swimmers nowadays with the age-related IPP of swimmers who have reached elite level. In this way, IPP might in addition to swim performance function as an additional tool for federations and coaches to further select and monitor future talented swimmers. However, at all times, federations and coaches should be aware that performance progression is not a linear process and that there are different pathways to elite level performance. Therefore, we want to emphasize to use IPP as one of many parameters which can provide insight about performance progression of talented swimmers.

## Data Availability Statement

Publicly available datasets were analyzed in this study. This data can be found here: www.swimrankings.net.

## Ethics Statement

The studies involving human participants were reviewed and approved by the Local Ethical Committee of the University Medical Center Groningen, University of Groningen, The Netherlands. Written informed consent from the participants' legal guardian/next of kin was not required to participate in this study in accordance with the national legislation and the institutional requirements.

## Author Contributions

AP wrote the manuscript and conducted all statistical analysis. AP and RK conducted the data collection, processing, and analysis. RK, IS, CV, and ME-G reviewed the concept and final manuscript. All authors contributed to the article and approved the submitted version.

## Conflict of Interest

The authors declare that the research was conducted in the absence of any commercial or financial relationships that could be construed as a potential conflict of interest.
