# Supplementary material for: Interim Performance Progression (IPP) During Consecutive Season Best Performances of Talented Swimmers
Source: Front Sports Act Living. 2020 Nov 3;2:579008. doi: 10.3389/fspor.2020.579008 (PMC7739762; doi:10.3389/fspor.2020.579008)
Supplement: Supplementary file 1 [file Table_1.docx]

| Supplementary Table 1: Performance benchmarks for identifying talented swimmers based on the performance development of elite swimmers investigated in previous research of Post et al. (2020). | | |
| --- | --- | --- |
| **Age** | **Males** | **Females** |
| 12 | 138.4 | 130.9 |
| 13 | 136.1 | 130.4 |
| 14 | 125.8 | 130.4 |
| 15 | 125.8 | 123.3 |
| 16 | 120.3 | 118.6 |
| 17 | 115.8 | 118.6 |
| 18 | 113.9 | 113.2 |
| 19 | 113.9 | 113.2 |
| 20 | 110.3 | 113.1 |
| 21 | 110.3 | 110.1 |
| 22 | 108.4 | 110.1 |
| 23 | 107.8 | 109.0 |
| 24 | 107.3 | 109.0 |
| 25 | 105.7 | 107.8 |
| 26 | 105.7 | 105.7 |

| Supplementary Table 2: Mean and SD for previous season best rST. first rST. current season rST. PP_A_ and PP_B_. per age. per best performance group of male swimmers. | | | | | | | |
| --- | --- | --- | --- | --- | --- | --- | --- |
| **Age** | **Best performance group** | **N** | **Previous season best rST** | **First rST** | **Current season rST** | **PP**_A_ | **PP_B_** |
| 14 | Elite | 7 | 118.8 ± 4.2 | 118.6 ± 4.9 | 114.9 ± 3.1 | 99.8 ± 1.9 | 18.8 ± 6.5 |
| 14 | High-competitive | 117 | 124.5 ± 5.2 | 121.7 ± 4.0 | 118.7 ± 3.7 | 97.8 ± 2.4^*^ | 13.6 ± 8.8 |
| 15 | Elite | 13 | 114.6 ± 5.1 | 113.6 ± 4.3 | 110.8 ± 3.5 | 99.2 ± 2.3 | 20.5 ± 9.9 |
| 15 | High-competitive | 260 | 119.8 ± 5.0 | 118.4 ± 4.3 | 115.9 ± 3.8 | 98.9 ± 2.3 | 13.0 ± 9.9^*^ |
| 16 | Elite | 22 | 109.8 ± 2.8 | 109.5 ± 2.7 | 107.0 ± 2.0 | 99.7 ± 1.2 | 25.1 ± 14.5 |
| 16 | High-competitive | 369 | 115.2 ± 3.6 | 115.5 ± 3.4 | 113.1 ± 3.1 | 100.3 ± 2.1 | 15.2 ± 12.1^*^ |
| 17 | Elite | 38 | 107.9 ± 2.4 | 108.0 ± 2.2 | 105.8 ± 1.9 | 100.1 ± 2.1 | 26.6 ± 17.3 |
| 17 | High-competitive | 452 | 112.9 ± 3.0 | 113.6 ± 2.9 | 111.2 ± 2.6 | 100.6 ± 2.0 | 16.7 ± 12.4^*^ |
| 18 | Elite | 61 | 106.4 ± 2.2 | 107.0 ± 2.4 | 105.4 ± 1.9 | 100.6 ± 1.5 | 22.1 ± 18.2 |
| 18 | High-competitive | 433 | 111.1 ± 2.7 | 112.3 ± 2.8 | 110.1 ± 2.3 | 101.1 ± 2.0 | 16.8 ± 12.8^*^ |
| 19 | Elite | 87 | 105.8 ± 2.1 | 106.6 ± 2.5 | 104.7 ± 1.9 | 100.7 ± 1.7 | 26.1 ± 20.0 |
| 19 | High-competitive | 451 | 110.8 ± 2.8 | 111.8 ± 3.0 | 109.7 ± 2.5 | 100.9 ± 1.8 | 16.5 ± 12.3^*^ |
| 20 | Elite | 93 | 104.9 ± 1.7 | 106.2 ± 2.0 | 104.3 ± 1.7 | 101.2 ± 1.5 | 29.4 ± 23.7 |
| 20 | High-competitive | 275 | 108.4 ± 1.9 | 109.6 ± 2.3 | 107.4 ± 1.6 | 101.1 ± 1.7 | 20.8 ± 14.8^*^ |
| 21 | Elite | 93 | 104.6 ± 1.4 | 105.7 ± 1.7 | 103.9 ± 1.4 | 101.0 ± 1.6 | 29.8 ± 22.1 |
| 21 | High-competitive | 273 | 108.2 ± 2.1 | 109.4 ± 2.3 | 107.4 ± 1.7 | 101.1 ± 1.7 | 19.8 ± 14.6^*^ |
| 22 | Elite | 73 | 104.5 ± 1.4 | 105.8 ± 1.9 | 103.7 ± 1.4 | 101.2 ± 1.4 | 34.8 ± 22.2 |
| 22 | High-competitive | 143 | 107.2 ± 1.5 | 108.5 ± 2.1 | 106.2 ± 1.2 | 101.2 ± 1.6 | 24.1 ± 15.5^*^ |
| 23 | Elite | 58 | 103.9 ± 1.3 | 105.5 ± 1.7 | 103.6 ± 1.5 | 101.5 ± 1.4 | 34.9 ± 23.1 |
| 23 | High-competitive | 98 | 106.9 ± 1.6 | 108.2 ± 1.8 | 105.9 ± 1.0 | 101.2 ± 1.6 | 25.4 ± 15.0^*^ |
| 24 | Elite | 38 | 104.2 ± 1.5 | 105.5 ± 1.3 | 103.5 ± 1.2 | 101.2 ± 1.3 | 34.3 ± 20.7 |
| 24 | High-competitive | 55 | 106.5 ± 1.3 | 108.0 ± 1.7 | 105.8 ± 1.0 | 101.5 ± 1.3 | 26.3 ± 14.1^*^ |
| ^* indicates significant difference (tested for PPa and PPb) between elite performance group. p<0.05^ | | | | | | | |

| Supplementary Table 3: Mean and SD for previous season best rST. first rST. current season rST. PP_A_ and PP_B_. per age. per best performance group of female swimmers. | | | | | | | |
| --- | --- | --- | --- | --- | --- | --- | --- |
| **Age** | **Best performance group** | **N** | **Previous season best rST** | **First rST** | **Current season rST** | **PP**_A_ | **PP_B_** |
| 12 | Elite | 7 | 120.3 ± 2.2 | 118.4 ± 2.0 | 114.6 ± 2.5 | 98.5 ± 1.4 | 20.6 ± 9.9 |
| 12 | High-competitive | 142 | 128.8 ± 7.7 | 125.5 ± 5.7 | 121.6 ± 4.4 | 97.6 ± 3.8 | 14.5 ± 11.1 |
| 13 | Elite | 22 | 115.1 ± 5.0 | 114.0 ± 4.2 | 111.0 ± 3.4 | 99.2 ± 2.4 | 21.1 ± 11.7 |
| 13 | High-competitive | 233 | 121.5 ± 4.7 | 120.1 ± 4.2 | 117.0 ± 3.6 | 98.9 ± 2.5 | 14.6 ± 10.1^*^ |
| 14 | Elite | 30 | 110.0 ± 3.6 | 110.3 ± 3.3 | 107.7 ± 2.3 | 100.3 ± 1.9 | 23.4 ± 14.5 |
| 14 | High-competitive | 389 | 117.0 ± 4.2 | 116.9 ± 3.8 | 114.6 ± 3.6 | 99.9 ± 2.3 | 13.4 ± 11.0^*^ |
| 15 | Elite | 73 | 107.4 ± 2.3 | 108.1 ± 2.7 | 106.2 ± 2.1 | 100.7 ± 1.9 | 23.0 ± 16.9 |
| 15 | High-competitive | 511 | 114.8 ± 3.7 | 115.6 ± 3.6 | 113.1 ± 3.2 | 100.7 ± 2.0 | 15.6 ± 11.3^*^ |
| 16 | Elite | 104 | 106.9 ± 2.1 | 107.9 ± 2.3 | 106.2 ± 1.8 | 100.9 ± 1.7 | 20.4 ± 15.1 |
| 16 | High-competitive | 549 | 113.4 ± 3.0 | 114.3 ± 3.1 | 112.1 ± 2.7 | 100.8 ± 1.9 | 14.7 ± 11.4^*^ |
| 17 | Elite | 120 | 106.5 ± 2.0 | 107.4 ± 2.0 | 105.9 ± 1.8 | 100.9 ± 1.3 | 20.0 ± 15.9 |
| 17 | High-competitive | 493 | 112.8 ± 3.0 | 114.0 ± 3.1 | 111.8 ± 2.8 | 101.1 ± 1.9 | 14.9 ± 11.4^*^ |
| 18 | Elite | 110 | 105.9 ± 1.7 | 106.6 ± 1.7 | 105.3 ± 1.7 | 100.7 ± 1.4 | 20.7 ± 17.9 |
| 18 | High-competitive | 297 | 111.0 ± 2.4 | 112.0 ± 2.4 | 110.1 ± 2.0 | 101.0 ± 1.7 | 15.3 ± 11.5^*^ |
| 19 | Elite | 121 | 105.6 ± 1.7 | 106.6 ± 2.2 | 105.1 ± 1.9 | 100.9 ± 1.4 | 22.1 ± 17.5 |
| 19 | High-competitive | 244 | 110.6 ± 2.3 | 111.7 ± 2.4 | 109.7 ± 1.9 | 101.0 ± 1.7 | 16.3 ± 11.6^*^ |
| 20 | Elite | 116 | 105.4 ± 1.9 | 106.3 ± 2.0 | 104.7 ± 1.7 | 100.9 ± 1.2 | 26.0 ± 21.5 |
| 20 | High-competitive | 193 | 110.5 ± 2.3 | 111.7 ± 2.6 | 109.4 ± 2.0 | 101.2 ± 1.6 | 18.6 ± 10.9^*^ |
| 21 | Elite | 107 | 105.0 ± 1.8 | 106.0 ± 2.0 | 104.4 ± 1.7 | 100.9 ± 1.5 | 27.1 ± 21.5 |
| 21 | High-competitive | 67 | 109.0 ± 1.6 | 110.0 ± 1.7 | 108.1 ± 1.1 | 100.9 ± 1.4 | 17.9 ± 11.5^*^ |
| 22 | Elite | 87 | 104.9 ± 1.7 | 105.8 ± 1.9 | 104.4 ± 1.6 | 100.9 ± 1.3 | 24.4 ± 18.2 |
| 22 | High-competitive | 47 | 109.2 ± 1.4 | 109.9 ± 1.8 | 108.0 ± 1.0 | 100.6 ± 1.5 | 17.5 ± 11.2^*^ |
| ^* indicates significant difference (tested for PPa and PPb) between elite performance group. p<0.05^ | | | | | | | |

| Supplementary Table 4. Test statistics of independent sample t-tests and Mann Whitney tests for talented male swimmers. | | | | | | | | | | |  |
| --- | --- | --- | --- | --- | --- | --- | --- | --- | --- | --- | --- |
| PP_A_ | | | | | | | | | | |  |
| Age | t or U | | *Df* | | p | | CI | | r or *d* | |  |
| 14^*^ | 217.00 | |  | | 0.037 | |  | | 0.18 | |  |
| 15^*^ | 1.537.00 | |  | | 0.582 | |  | | 0.03 | |  |
| 16^*^ | 4.698.00 | |  | | 0.215 | |  | | 0.06 | |  |
| 17 | -1.536 | | 488 | | 0.125 | | [-1.1 - 0.15] | | -0.26 | |  |
| 18 | -1.775 | | 492 | | 0.076 | | [-0.99 - 0.05] | | -0.24 | |  |
| 19 | -1.040 | | 536 | | 0.299 | | [-0.62 - 0.19] | | -0.12 | |  |
| 20 | 0.445 | | 366 | | 0.657 | | [-0.30 -0.47] | | 0.05 | |  |
| 21 | -0.824 | | 364 | | 0.410 | | [-0.57 - 0.23] | | -0.10 | |  |
| 22 | 0.212 | | 214 | | 0.832 | | [-0.40 - 0.43] | | 0.03 | |  |
| 23 | 1.248 | | 154 | | 0.214 | | [-0.18 - 0.81] | | 0.14 | |  |
| 24 | -0.889 | | 91 | | 0.376 | | [-0.79 - 0.30] | | -0.14 | |  |
| PP_B_ | | | | | | | | | | |  |
| Age | t or U | | *Df* | | p | | CI | | r or *d* | |  |
| 14^*^ | 124.00 | |  | | 0.113 | |  | | 0.14 | |  |
| 15^*^ | 978.00 | |  | | 0.010 | |  | | 0.15 | |  |
| 16^*^ | 2.401.00 | |  | | 0.001 | |  | | 0.16 | |  |
| 17 | 3.433 | | 40.249 | | 0.001 | | [4.0 - 15.6] | | 0.77 | |  |
| 18 | 2.180 | | 68.567 | | 0.033 | | [0.45 -10.1] | | 0.39 | |  |
| 19 | 4.345 | | 98.815 | | <0.001 | | [5.3 - 14.1] | | 0.70 | |  |
| 20 | 3.283 | | 117.111 | | 0.001 | | [3.4 - 13.8] | | 0.49 | |  |
| 21 | 4.064 | | 120.361 | | <0.001 | | [5.1 - 14.9] | | 0.59 | |  |
| 22 | 3.685 | | 108.826 | | <0.001 | | [5.0 - 16.5] | | 0.59 | |  |
| 23 | 2.805 | | 85.717 | | 0.006 | | [2.8 - 6.3] | | 0.03 | |  |
| 24 | 2.058 | | 60.276 | | 0.044 | | [0.23 -15.7] | | 0.03 | |  |
| ^* For age 14 through 16. Mann Whitney tests were performed instead of independent sample t-tests (test statistic is reported U and effect size is r)^ | | | | | | | | | | |  |
| Supplementary Table 5. Test statistics of independent sample t-tests and Mann Whitney tests for talented female swimmers. | | | | | | | | | | | |
| PP_A_ | | | | | | | | | | | |
| Age | | t or U | | *Df* | | p | | CI | | r or *d* | |
| 12^*^ | | 455 | |  | | 0.706 | |  | | 0.03 | |
| 13^*^ | | 2.420 | |  | | 0.665 | |  | | 0.03 | |
| 14 | | 0.989 | | 417.000 | | 0.323 | | [-0.42 - 1.30] | | 0.19 | |
| 15 | | -2.390 | | 582.000 | | 0.811 | | [-0.56 - 0.44] | | -0.03 | |
| 16 | | 0.431 | | 651.000 | | 0.667 | | [0.30 - 0.47] | | 0.05 | |
| 17 | | -1.052 | | 611.000 | | 0.293 | | [-0.58 - 0.17] | | -0.11 | |
| 18 | | -1.487 | | 405.000 | | 0.138 | | [-0.64 - 0.09] | | -0.17 | |
| 19 | | -4.010 | | 363.000 | | 0.688 | | [-0.42 - 0.28] | | -0.04 | |
| 20 | | -1.612 | | 307.000 | | 0.108 | | [-0.63 - 0.06] | | -0.19 | |
| 21 | | 0.081 | | 172.000 | | 0.935 | | [-0.43 - 0.47] | | 0.01 | |
| 22 | | 1.380 | | 132.000 | | 0.170 | | [-0.15 - 0.84] | | 0.25 | |
| PP_B_ | | | | | | | | | | | |
| Age | | t or U | | *Df* | | p | | CI | | r or *d* | |
| 12^*^ | | 318.00 | |  | | 0.110 | |  | | 0.13 | |
| 13^*^ | | 1.743.00 | |  | | 0.013 | |  | | 0.15 | |
| 14 | | 3.712 | | 31.633 | | 0.001 | | [4.5 - 15.5 ] | | 0.89 | |
| 15 | | 3.632 | | 81.476 | | <0.001 | | [3.4 - 11.5] | | 0.61 | |
| 16 | | 3.677 | | 126.220 | | <0.001 | | [2.6 - 8.8] | | 0.47 | |
| 17 | | 3.318 | | 150.036 | | 0.001 | | [2.1 - 8.1] | | 0.41 | |
| 18 | | 2.950 | | 143.183 | | 0.004 | | [1.8 - 9.0] | | 0.40 | |
| 19 | | 3.281 | | 172.146 | | 0.001 | | [2.3 - 9.3] | | 0.42 | |
| 20 | | 3.427 | | 151.112 | | 0.001 | | [3.1 - 11.6] | | 0.47 | |
| 21 | | 3.662 | | 168.445 | | <0.001 | | [4.2 - 14.1] | | 0.50 | |
| 22 | | 2.703 | | 129.481 | | 0.008 | | [1.8 - 11.9] | | 0.43 | |
| ^* For age 12 through 13. Mann Whitney tests were performed instead of independent sample t-tests (test statistic is U and effect size is r)^ | | | | | | | | | | | |
